# Attention-deficit/hyperactivity disorder medication use and cardiometabolic conditions in pregnancy: a population-based cohort study

**DOI:** 10.1007/s00737-025-01664-0

**Published:** 2026-03-03

**Authors:** Andrea Sit, Claudia Bruno, Masako Araki, Malcolm B. Gillies, Helga Zoega

**Affiliations:** 1 School of Population Health, Faculty of Medicine and Health, UNSW Sydney, Sydney, Australia; 2 Centre of Public Health Sciences, Faculty of Medicine, University of Iceland, Reykjavik, Iceland

**Keywords:** ADHD, Stimulants, Pregnancy, Gestational hypertension, Gestational diabetes mellitus

## Abstract

**Purpose:**

Use of medications to treat attention-deficit/hyperactivity disorder (ADHD) is increasingly prevalent among women of reproductive age, but little is understood about their potential cardiometabolic effects in pregnancy. We aimed to examine associations between ADHD medication use and cardiometabolic conditions during pregnancy (gestational hypertension, preeclampsia, and gestational diabetes) and the pharmacological treatment thereof.

**Methods:**

Linking statewide hospital and dispensing data, we conducted a population-based matched cohort study of women who gave birth between January 2014 and June 2021 in New South Wales, Australia (*n* = 312,697). We compared the incidence of cardiometabolic conditions and cardiometabolic medication use during pregnancy among women who used ADHD medications during pregnancy (*n* = 336) with a 1:10 matched cohort of unexposed women, and with women who used ADHD medications in the 12 months before pregnancy (*n* = 252). We used Poisson regression models to calculate risk ratios (RR) and 95% confidence intervals (CI), adjusting for sociodemographic and pregnancy-related factors.

**Results:**

Compared with unexposed women, women who used ADHD medications during pregnancy had an increased risk of gestational hypertension (adjusted RR: 1.76, 95% CI: 1.20–2.57) and gestational diabetes (aRR: 1.41, 95% CI: 1.09–1.82), with slightly elevated risk estimates for preeclampsia (aRR: 1.30, 95% CI: 0.82–2.05) and cardiometabolic medication use (aRR: 1.40, 95% CI: 0.97–2.01). Compared with women who used ADHD medications before pregnancy only, risk estimates attenuated for all outcomes except gestational diabetes (aRR: 1.76, 95% CI: 1.06–2.93).

**Conclusions:**

Women using ADHD medications had an elevated incidence of cardiometabolic conditions during pregnancy, but it remains unclear to what extent this is attributable to medications rather than the underlying ADHD.

**Supplementary information:**

The online version contains supplementary material available at 10.1007/s00737-025-01664-0.

## Article highlights

- There is limited evidence regarding the cardiometabolic effects of ADHD medication use during pregnancy.
- In this population-based cohort study, we found that pregnant women who use ADHD medications may be at greater risk of gestational hypertension, preeclampsia and gestational diabetes.
- It is unclear whether or to what extent this represents an effect ofADHD medication as opposed to the underlying ADHD.

**Supplementary information:**

The online version contains supplementary material available at 10.1007/s00737-025-01664-0.

## Introduction

Affecting 8.2% of children and 2.5% of adults in Australia (Australian 2024), attention-deficit/hyperactivity disorder (ADHD) is a common neurodevelopmental condition associated with substantial burden in the form of psychiatric comorbidity and/or impairment across educational, vocational and social domains (Thomas et al. 2015; Posner et al. 2020). Despite increasing awareness of the condition in recent decades, it remains under-diagnosed and undertreated, especially among females (Young et al. 2020). During childhood, the prevalence of ADHD among boys is 2- to 9-fold higher than in girls, both in clinical and epidemiological samples; this ratio evens out in adulthood, likely reflecting the under-recognition and later onset of ADHD in females (Quinn and Madhoo 2014).

Both in Australia and worldwide, the use of pharmacotherapy for ADHD has at least doubled in the last decade among children and adults, with the most rapid increases in use among young women of reproductive age (Bruno et al. 2023; Raman et al. 2018). Though it appears that most women at least temporarily suspend their use of ADHD medications during pregnancy, prescribing rates among pregnant women are also on the rise (Poulton et al. 2018; Srinivas et al. 2023; Cohen et al. 2023; Louik et al. 2015; Anderson et al. 2020); between 2010 and 2019, ADHD medication use increased in Norway from 2.5 to 5.5/1000 pregnancies and in Sweden from 1.6 to 7.9/1000 pregnancies (Cohen et al. 2023). Despite these trends, there remains limited data as to the safety profile of these medications, including stimulants and non-stimulants, during pregnancy (Therapeutic 2023; Li et al. 2020).

Existing literature suggests an association between long-term use of ADHD medications and increased risk of cardiometabolic disease, including hypertension and arterial disease (Zhang et al. 2024). Likewise, in the pregnant population, there is mixed evidence of an association between exposure to ADHD medications and elevated risk of cardiometabolic conditions during pregnancy. A recent pooled meta-analysis reported that ADHD medication use during pregnancy was not associated with pregnancy-related complications including gestational hypertension, preeclampsia and gestational diabetes (Li et al. 2020). On the other hand, several large pharmacoepidemiological studies have found that ADHD medication exposure during pregnancy may correlate with increased risk of preeclampsia (Poulton et al. 2018; Camacho et al. 2023; Cohen et al. 2017; NSW 2018). However, it is not known to what extent these findings represent causality or are otherwise attributable to confounding factors, such as the underlying ADHD. A large, matched-cohort study by Walsh and colleagues found that women with ADHD had moderately elevated odds of gestational hypertension, preeclampsia, gestational diabetes and other obstetric complications; these risks were elevated in both medicated and unmedicated women, suggesting that the underlying ADHD may contribute to adverse cardiometabolic outcomes in pregnancy, independent of medication use (Centre 2021).

In view of the rising prevalence of stimulant and non-stimulant medication use for the treatment of ADHD, and the limitations of the current evidence, more comprehensive research is warranted to better understand the safety and potential implications of their use during pregnancy. Further evidence will inform clinical practice guidelines about medication safety and, ultimately, guide decision-making by clinicians and women during pregnancy, such as whether to discontinue or change treatment to optimise maternal and fetal health outcomes.

Leveraging a linkage of two Australian population-based data collections recording hospital and medication dispensing data, our study explored ADHD medication use and cardiometabolic conditions arising in pregnancy. More specifically, we aimed to estimate the incidence of cardiometabolic conditions in pregnancy (including gestational hypertension, preeclampsia and gestational diabetes), and initiation of pharmacological treatment thereof, among women who used ADHD medications during pregnancy and those who did not use ADHD medications during pregnancy. We further examined potential associations between ADHD medication use in pregnancy and pregnancy-related cardiometabolic conditions, as well as initiation of cardiometabolic medication, accounting for women´s sociodemographic, health- and pregnancy-related characteristics.

## Methods

### Study design & data sources

We conducted a population-based matched cohort study based on linkage of the New South Wales (NSW) Admitted Patient Data Collection (APDC) and Pharmaceutical Benefits Scheme (PBS) dispensing data in Australia. The Australian universal healthcare system and health insurance scheme, known as Medicare, entitles access to subsidised health services, including prescription medications and treatment in hospitals, for all citizens and permanent residents. The APDC is a statutory data collection containing records of all hospital admissions to NSW public and private hospitals and day procedure centres, with diagnoses coded according to the International Classification of Diseases, Version 10, Australian Modification (ICD-10-AM) and procedures coded according to the Australian Classification of Health Interventions (ACHI) (Mellish et al. 2015). Almost all Australian mothers give birth in a hospital labour ward or birth centre (Zoega et al. 2024). PBS dispensing data capture all subsidised medications dispensed nationwide, excluding those dispensed during public hospital inpatient stays and on discharge from public hospitals (WHO 2024). These two data collections were accessed in the Medicines Intelligence (MedIntel) Data Platform, an enduring population-based linked data platform pertaining to all Medicare-eligible adults who were recorded as residing in NSW anytime between 2005 and 2020 (Baldwin et al. 2021). The present study used PBS and APDC records from 1 January 2004 to 30 June 2021.

### Study population

Our study included adult women (aged ≥ 18 years and < 55 years) residing in NSW who had their first birth in NSW between 1 January 2014 and 30 June 2021. We considered women to have given birth if they had a hospital admission with a diagnosis or procedure code relating to delivery (Online Resource 1). If women had multiple hospital admissions, we included only the first delivery record observed within a 10-year lookback period. Women who had prior dispensing of cardiometabolic medications in the 12 months before pregnancy start were excluded from all analyses to confirm new use of these medications during pregnancy.

### ADHD medication use

Our exposure of interest was ADHD medication use during pregnancy. We used the World Health Organization Anatomical Therapeutic Chemical (ATC) codes to identify ADHD medications (Pratt et al. 2018) that were PBS-listed in Australia and approved for the treatment of ADHD during the study period, including stimulants, methylphenidate (N06BA04), lisdexamfetamine (N06BA12) and dexamfetamine (N06BA02 with authority code for ADHD), and non-stimulants atomoxetine (N06BA09) and guanfacine (C02AC02).

We classified women who gave birth into three mutually exclusive groups based on timing of exposure to ADHD medications relative to their pregnancy period (Online Resource 2). The APDC does not report specific pregnancy details such as date of delivery; as such, we used the date of admission to hospital as a proxy for date of delivery. Pregnancy start was estimated as the delivery date minus 40 weeks, or for those with a diagnosis code indicating preterm birth, pregnancy start was estimated using an ICD-10-AM code for gestational age at delivery (Online Resource 3). Exposure was classified into the following categories:

1. *During pregnancy*, defined as having any dispensing of ADHD medication between pregnancy start and delivery.
2. *Before pregnancy*, defined as women with a dispensing of ADHD medications from 12 months to 45 days before pregnancy start, with no dispensing from 45 days before pregnancy start until date of delivery; and.
3. *Unexposed*, defined as no record of a dispensing of ADHD medications in the 12 months before pregnancy start nor during pregnancy. Women who used ADHD medication *during pregnancy* were 1:10 matched at random by calendar year and maternal age at delivery with the *unexposed* group.

### Cardiometabolic outcomes

Our main outcome measures were cardiometabolic conditions arising during pregnancy and new use of cardiometabolic medications during pregnancy. We identified cardiometabolic conditions arising during pregnancy according to ICD-10-AM diagnosis codes for gestational hypertension, preeclampsia, preeclampsia superimposed on chronic hypertension, eclampsia, and diabetes mellitus arising during pregnancy (Online Resource 4) (Beech and Mangos 2021). New use of cardiometabolic medications was ascertained by a first dispensing of any antihypertensive and/or antihyperglycaemic medications during pregnancy, that is, no dispensing of such medications in the 12 months leading to pregnancy; these medications were identified by relevant ATC codes (Online Resource5) (Fox et al. 2019; Laurie and McIntyre 2020).

### Sociodemographic- and pregnancy-related characteristics

We examined several sociodemographic and pregnancy-related covariates for adjustment that we considered potential confounders, as they have previously been established as risk factors for the development of hypertensive disorders and/or diabetes in pregnancy (Zakiyah et al. 2018; Park et al. 2018). We extracted sociodemographic information from the hospitalisation record of the delivery, including calendar year of delivery, maternal age, maternal country of birth, remoteness (as classified by the Australian Statistical Geography Standard (ASGS)), Index of Relative Socioeconomic Disadvantage (IRSD), and hospital type (public or private). Pregnancy-related characteristics, including pre-existing hypertension and diabetes, multiple gestation, and smoking during pregnancy were determined based on the relevant ICD-10-AM codes (Online Resource4). We also adjusted for use of other psychotropic medications during pregnancy, including opioid analgesics (N02A), antiepileptics (N03A), antipsychotics (N05A), anxiolytics, hypnotics, sedatives (N05B and N05C), and antidepressants (N06A), as these may be associated with cardiometabolic conditions during pregnancy due to their metabolic side effects (Frayne et al. 2021; Heinonen et al. 2022; R 2021; von Elm et al. 2008). We also reported the prevalence of use of other psychotropic medications in the 12 months before pregnancy.

### Data analysis

First, we estimated the prevalence of ADHD medication use among women during their first pregnancy. In our main analysis, we compared the incidence of cardiometabolic conditions and cardiometabolic medication use during pregnancy among women who used ADHD medications *during pregnancy*, with women who were *unexposed*. Women who used ADHD medication *during pregnancy* were 1:10 matched at random by calendar year and maternal age at delivery with the *unexposed* group. To mitigate the potential for confounding by underlying ADHD indication, we then performed a secondary analysis, comparing women who used ADHD medications *during pregnancy*, with those who had used them in the 12 months *before pregnancy.*

To assess the association between ADHD medication exposure and cardiometabolic conditions arising in pregnancy, we used Poisson regression models to calculate risk ratios (RR). We presented a crude model (adjusted for only the matching variables of maternal age and calendar year of delivery), and an adjusted model (adjusted for the matching variables of maternal age and calendar year of delivery, plus the potential confounding variables of maternal country of birth, remoteness, socioeconomic disadvantage, hospital type, smoking during pregnancy, pre-existing hypertension or diabetes, and other psychotropic medication use). We used robust standard errors to estimate the 95% confidence intervals. We conducted a complete-case analysis, excluding 0.02% (*n* = 68) of records with missing information on remoteness and socioeconomic disadvantage (Online Resource 6). There were no other missing observations for the variables used for matching or adjustment.

All statistical analyses were performed in R (version 4.3.0) (Weisler et al. 2005). This observational study was reported in accordance with the Strengthening the Reporting of Observational Studies in Epidemiology (STROBE) Statement guidelines (Online Resource7) (Andrade 2018).

### Ethics

This study received ethical approval from the Australian Institute of Health and Welfare (AIHW) Human Research Ethics Committee (HREC) (approval number EO2021/1/1233) and the NSW Population and Health Services Research Ethics Committee (PHSREC) (approval number 2020/ETH02273). Direct access to the data and analytical files to other individuals or authorities is not permitted without the express permission of the approving human research ethics committees and data custodians.

## Results

We identified a total of 329,358 women who gave birth during the study period, of whom 370 (0.11%) were dispensed ADHD medications during their first observed pregnancy. Of these, 163 (48.5%) used dexamfetamine, 139 (41.4%) methylphenidate, 27 (8.0%) lisdexamfetamine, 12 (3.6%) atomoxetine and < 6 (< 1.8%) guanfacine. The prevalence of ADHD medication use during pregnancy increased over the study period, rising from 0.09% in 2014–2015 to 0.19% in 2020–2021. Compared to the *unexposed* group, women who used ADHD medication during pregnancy were more likely to be Australian-born (87.8% vs. 63.4%), and to smoke during pregnancy (10.4% vs. 6.5%), and to use other psychotropic medications during pregnancy (44.0% vs. 11.5%) and in the 12 months before pregnancy (61.0% vs. 19.5%) (Table 1).

**Table 1:** Sociodemographic- and pregnancy-related characteristics of the study population according to ADHD medication use

	ADHD medication use during pregnancy *N* (%)		Unexposed (prior to matching)^a^ *N* (%)		ADHD medication use before pregnancy *N* (%)	
Total, *N*	336		312,050		252	
Year of childbirth, *n* (%)						
2014-15	73	(21.7%)	86,638	(27.7%)	61	(24.2%)
2016-17	80	(23.8%)	86,688	(27.8%)	69	(27.4%)
2018-19	83	(24.7%)	82,855	(26.6%)	72	(27.6%)
2020-21	100	(29.8%)	55,869	(17.9%)	50	(19.9%)
Maternal age at childbirth (years), mean (SD)	29.1 (6.2)		30.4 (5.5)		26.8 (7.1)	
Maternal age, years						
18–24	91	(27.1%)	53,899	(17.3%)	118	(46.8%)
25–29	95	(28.3%)	88,307	(28.3%)	50	(19.8%)
30–34	87	(25.9%)	106,651	(34.2%)	44	(17.5%)
≥ 35	63	(18.8%)	63,193	(20.3%)	40	(15.9%)
Maternal country of birth						
Australia	295	(87.8%)	183,805	(58.9%)	223	(88.5%)
Overseas	41	(12.2%)	128,245	(41.1%)	29	(11.5%)
Remoteness						
Major cities	261	(77.7%)	252,757	(81.0%)	173	(68.7%)
Inner regional	58	(17.3%)	44,936	(14.4%)	68	(27.0%)
Outer regional/Remote	17	(5.1%)	14,357	(4.6%)	11	(4.4%)
SEIFA						
1 – most disadvantaged	65	(19.3%)	67,037	(21.5%)	64	(25.4%)
2	62	(18.5%)	55,857	(17.9%)	40	(15.9%)
3	52	(15.5%)	62,440	(20.0%)	51	(20.2%)
4	77	(22.9%)	66,014	(21.2%)	49	(19.4%)
5 – least disadvantaged	80	(23.8%)	60,702	(19.5%)	48	(19.0%)
Hospital type						
Public	277	(82.4%)	242,299	(77.6%)	212	(84.1%)
Private	59	(17.6%)	69,751	(22.4%)	40	(15.9%)
Marital status						
Married	219	(65.2%)	263,192	(84.3%)	149	(59.1%)
Unmarried	113	(33.6%)	46,860	(15.0%)	100	(39.7%)
Missing	4	(1.2%)	1,998	(0.6%)	3	(1.2%)
Multiple gestation	< 6	(< 1.8%)	4,623	(1.5%)	< 6	(< 2.4%)
Smoking during pregnancy	35	(10.4%)	15,965	(5.1%)	40	(15.9%)
Pre-existing hypertension	< 6	(< 1.8%)	578	(0.2%)	0	(0%)
Pre-existing diabetes	0	(0%)	621	(0.2%)	< 6	(< 2.4%)
Use of any other psychotropic medication during pregnancy ^b^	148	(44.0%)	32,596	(10.4%)	79	(31.3%)
Antidepressants	114	(33.9%)	15,721	(5.0%)	59	(23.4%)
Antipsychotics	24	(7.1%)	1,638	(0.5%)	13	(5.2%)
Antiepileptics	10	(3.0%)	1,205	(0.4%)	< 6	(< 2.4%)
Opioids	41	(12.2%)	16,839	(5.4%)	28	(11.1%)
Use of any other psychotropic medication within 12 months prior to pregnancy	205	(61.0%)	59,011	(18.9%)	137	(54.4%)
Antidepressants	150	(44.6%)	26,277	(8.4%)	103	(40.9%)
Antipsychotics	34	(10.1%)	2,638	(0.8%)	38	(15.1%)
Antiepileptics	16	(4.8%)	1,951	(0.6%)	12	(4.8%)
Opioids	87	(25.9%)	32,748	(10.5%)	59	(23.4%)

^a^ Overall unexposed group, prior to matching. Characteristics of the matched group are presented in Online Resource 8

^b^ Psychotropic medication categories not mutually exclusive

ADHD, attention-deficit/hyperactivity disorder; SD, standard deviation; SEIFA, Socio-Economic Indexes for Areas

### Main analysis

**Fig. 1:**
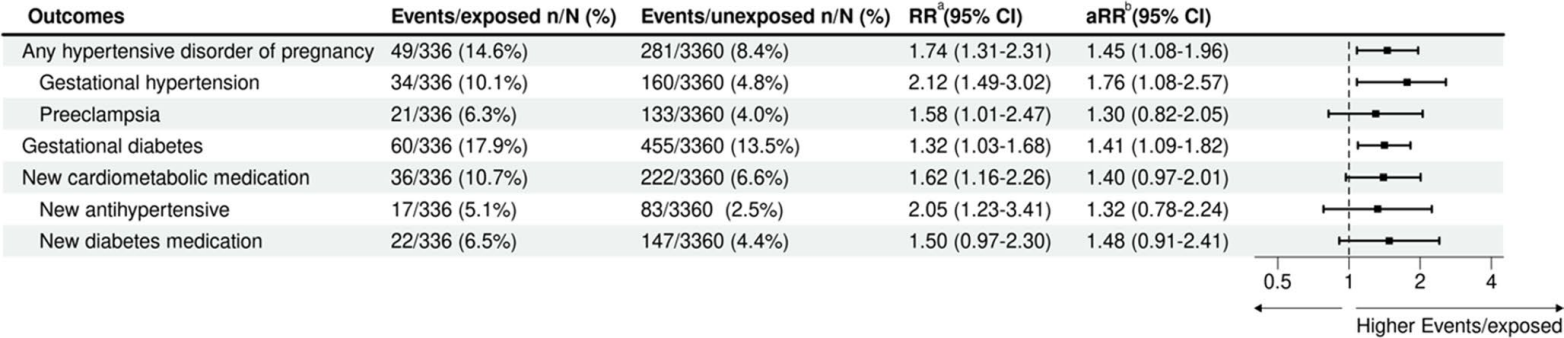
Risk ratios of cardiometabolic conditions arising in pregnancy in women with ADHD medication use during pregnancy relative to women unexposed to ADHD medications. ^a^ Adjusted for matching variables only: maternal age category and calendar year of delivery. ^b^ Fully adjusted model adjusted for: maternal age category, calendar year of delivery, maternal country of birth, remoteness, SEIFA, hospital type, pre-existing hypertension and diabetes, smoking during pregnancy, and other psychotropic use during pregnancy. ADHD, attention-deficit hyperactivity disorder; RR, risk ratio; CI, confidence interval; aRR, adjusted risk ratio; SEIFA, Socio-Economic Indexes for Areas

We found that women who used ADHD medication during pregnancy had elevated risk estimates for cardiometabolic conditions compared with the *unexposed* group; when we adjusted for additional confounders, all outcomes attenuated, with the exception of gestational diabetes. Compared with the *unexposed* group, women who used ADHD medication during pregnancy had a higher risk of gestational hypertension (10.1% vs. 4.8%; RR: 2.12, 95% CI: 1.49–3.02; adjusted RR (aRR): 1.76, 95% CI: 1.08–2.57) and gestational diabetes (17.9% vs. 13.5%; RR: 1.32 95% CI: 1.03–1.68; aRR: 1.41, 95% CI: 1.09–1.82). They also had an elevated risk estimate for preeclampsia (6.3% vs. 4.0%; RR: 1.58, 95% CI: 1.01–2.47; aRR: 1.30, 95% CI: 0.82–2.05), though confidence intervals indicate uncertainty around this estimate. Women who used ADHD medications during pregnancy also had an elevated risk estimate for new use of cardiometabolic medications during pregnancy (10.7% vs. 6.6%; RR: 1.62, 95% CI: 1.16–2.26; aRR: 1.40, 95% CI: 0.97–2.01) (Fig. 1).

### Secondary analysis

**Fig. 2:**
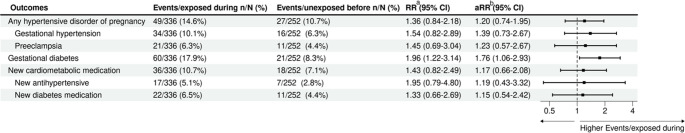
Risk ratios of cardiometabolic conditions arising in pregnancy in women with ADHD medication use during pregnancy relative to women with ADHD medication use in the 12 months before but not during pregnancy. ^a^ Adjusted for matching variables only: maternal age category and calendar year of delivery. ^b^ Fully adjusted model adjusted for: maternal age category, calendar year of delivery, maternal country of birth, remoteness, SEIFA, hospital type, pre-existing hypertension and diabetes, smoking during pregnancy, and other psychotropic use during pregnancy. ADHD, attention-deficit hyperactivity disorder; RR, risk ratio; CI, confidence interval; aRR, adjusted risk ratio; SEIFA, Socio-Economic Indexes for Areas

As a secondary comparison, we identified 252 women who used ADHD medication in the 12 months before but not during pregnancy. These women were younger on average than those who continued to use ADHD medication during pregnancy, but they were otherwise similar in their demographic and comorbidities (Table 1). When comparing women who used ADHD medication during pregnancy with women to those who used ADHD medication before pregnancy only, we again detected an elevated risk estimate for gestational diabetes, though the confidence interval was wide (aRR: 1.76, 95% CI: 1.06–2.93). There was, however, no robust evidence of an association with any hypertensive disorder of pregnancy, including gestational hypertension and preeclampsia, nor of an association with new use of cardiometabolic medications during pregnancy (Fig. 2).

## Discussion

In this population-based cohort study, we observed that women treated for ADHD may be at greater risk of new onset cardiometabolic conditions during pregnancy, but we were unable to infer whether, or to what extent, this represents an effect of ADHD medication as opposed to the underlying ADHD. In our main comparison, point estimates for all cardiometabolic outcomes during pregnancy (gestational hypertension, preeclampsia, gestational diabetes and initiation of antihypertensive and/or antihyperglycaemic medications) were modestly but consistently elevated, with wide confidence intervals due to small sample sizes. All estimates attenuated further in our secondary comparison; relative to women who had used ADHD medications before pregnancy, women who used ADHD medications during pregnancy had a slightly greater risk of gestational diabetes, but not of hypertensive disorders of pregnancy nor initiation of cardiometabolic medications. Taken together, our findings suggest that underlying ADHD and associated confounding factors may play a role in the elevated incidence of cardiometabolic observed among women using ADHD medication in pregnancy.

We found that women who used ADHD medications during pregnancy had a 1.7-fold increased risk of gestational hypertension (absolute risk difference = 5.3%) and a 1.3-fold increased risk of preeclampsia (absolute risk difference = 2.3%) compared with women who did not use these medications during pregnancy. This largely aligns with existing literature which offers mixed evidence of a relationship between ADHD medication use in pregnancy and risk of gestational hypertensive disorders such as preeclampsia. Most previous studies have focussed on maternal use of stimulant medications; it is hypothesised that stimulants may increase cardiometabolic risk via long-term sympathomimetic effects on the vasculature, for example, vasoconstrictive effects which may contribute to placental insufficiency in preeclampsia (Khajehei and Assareh 2020; Mellström et al. 2020). A recent study was inconclusive in its analysis of stimulant use in pregnancy and preeclampsia risk across women in Canada (OR (odds ratio): 2.02, 95% CI: 1.42–2.88) and NSW (OR: 1.50, 95% CI 0.77–2.94) (Camacho et al. 2023). The study’s NSW cohort was restricted to social security beneficiaries, and findings may not generalise to the broader population. Another population-based study of women in NSW by Poulton and colleagues found that stimulant exposure both prior to and during pregnancy was associated in a slight increase in the risk of preeclampsia (OR: 1.2, 95% CI:1.0–1.4.0.4; OR: 1.5, 95% CI: 0.8, 2.6) (Poulton et al. 2018). However, given that these findings were observed even in women who were treatment-free for several years prior to conception, the authors concluded that any association was not likely a direct effect of stimulant medication, but rather other factors associated with having ADHD.

Interestingly we observed that ADHD medication use during pregnancy was associated with a greater risk of gestational diabetes in both analyses. In contrastPoulton et al. found no association between ADHD medication use and gestational diabetes, based on data extracted from the NSW Perinatal Data Collection between 1994 and 2012 (Poulton et al. 2018). Rates of gestational diabetes reported in their study were considerably lower among both women with and without ADHD medication use than observed in our data; this likely reflects increases over time in the reporting and ascertainment of gestational diabetes in routine data collections in most countries, due to the introduction of screening programs and changing diagnostic criteria (Beech and Mangos 2021; Li et al. 2020). Nevertheless, our findings are somewhat unexpected, given that stimulant medications are known to suppress appetite and reduce overall overweight and obesity, a metabolic risk factor for the development of insulin resistance and diabetes (Nankervis et al. 2018). It is possible that the relationship we observed is the effect of unmeasured confounders, such as obesity, diet and other lifestyle factors, as suggested by the lack of attenuation for the outcome of gestational diabetes in our fully adjusted model. Furthermore, in our data we were unable to age-match women who used ADHD medication*during pregnancy* and *before* pregnancy, due to limited numbers. Women who continued to use ADHD medications *during pregnancy*were older on average, and this underlying difference could have exaggerated the effect estimate for gestational diabetes in our secondary comparison since maternal age is an independent risk factor for the condition and our age adjustment was based on broad age bands (Bruno et al. 2023).

Despite having an elevated incidence of hypertensive and diabetic conditions during pregnancy, we did not find conclusive evidence that women who used ADHD medications were more likely to commence medications to treat these conditions during pregnancy. This may be because pharmacotherapy is not the mainstay of treatment for gestational hypertension and gestational diabetes; rather, first line management is conservative, such as increased monitoring or lifestyle interventions (Zakiyah et al. 2018). The severity of these conditions might not have necessitated pharmacological treatment, for example as suggested by the stronger association with gestational hypertension compared to preeclampsia.

Whilst ours is the first Australian study to investigate non-stimulant medication use and cardiometabolic outcomes in the setting of pregnancy, the numbers were too small to conduct separate analyses of women who used them. This highlights the challenges of pregnancy research, where clinical trial data is scarce due to concerns of ethics and feasibility. Despite increasing ADHD medication use among Australian women, even population-based pharmacoepidemiological studies such as ours are limited by small numbers. In a study of Medicaid-eligible women in the United States, stimulant use in early pregnancy was associated with a 1.3 times greater risk of preeclampsia, whilst atomoxetine use did not appear to be associated with preeclampsia (Cohen et al. 2017). As newer non-stimulant treatments for ADHD emerge, more research regarding the safety of individual ADHD medications is warranted to determine if the risk of complications differs between stimulants and non-stimulants. Such data would inform treatment guidelines and assist expectant mothers and their clinicians in decision-making around ADHD medication management in the perinatal period.

### Strengths & Limitations

Our study is novel in its recent timespan between the years 2014 and 2021, capturing an era of increased recognition of ADHD in women and rising use of ADHD medications, including expanding access to lisdexamfetamine and non-stimulants in Australia (Bruno et al. 2023). Our use of statewide population-based datasets minimises potential sources of bias, such as recall bias and selection bias, and increases the generalisability of our findings.

Our study was also subject to several limitations. First is the potential for confounding by indication, whereby the association observed in our main analysis may have been influenced by the underlying condition of ADHD for which the medications were prescribed. To mitigate this, we performed a secondary comparison with women who used ADHD medication use before pregnancy, where the results were attenuated, though this was also limited by small numbers and lack of matching. Second, there is a potential for residual confounding even after matching and adjusting for several important confounding variables. We observed that women who used ADHD medications during pregnancy were older and were more likely to smoke and use of other psychotropic medications during pregnancy – characteristics consistent with previously reported patterns of ADHD treatment in the perinatal period (Srinivas et al. 2023). Some known cardiometabolic risk factors such as obesity, alcohol and substance use, and other lifestyle behaviours are not captured well in our data and therefore not accounted for in our analysis. We were also unable to measure and adjust for ADHD severity and duration of ADHD medication use. Third, the routine hospital and dispensing data used in this study may be inaccurate or incomplete in some respects. For example, whilst we accounted for smoking during pregnancy, this is likely an underestimate as it relies on being recorded during a hospital visit. There is also the potential for under-capture of private prescriptions, such as women dispensed lisdexamfetamine privately prior to 2021 who would be misclassified in the*unexposed* group. Fourth, we excluded women from all analyses if they had prior dispensing of cardiometabolic medications in the 12 months before pregnancy. Whilst analysing cardiometabolic conditions arising in pregnancy and new use of cardiometabolic medication preserves the internal validity of our study, this approach does not allow us to detect worsening of pre-existing cardiometabolic conditions following ADHD medication use. Finally, a dispensing of an ADHD medication may not correspond to actual use, particularly in the context of a pregnant study population. Women may have been dispensed medications and subsequently chosen not to take them out of caution upon discovering their pregnancy, especially in the absence of comprehensive safety data. We defined exposure based on at least one dispensing but requiring multiple dispensings may better reflect sustained use. In this case, our results may have underestimated the true association between ADHD medication exposure and cardiometabolic outcomes during pregnancy.

## Conclusions

In this population-based study, we observed that women who used ADHD medications during pregnancy had a modestly elevated incidence of cardiometabolic conditions during pregnancy (gestational hypertension, preeclampsia, gestational diabetes and initiation of antihypertensive and/or antihyperglycaemic medications). However, confidence intervals were wide due to small sample sizes, and estimates attenuated further in our secondary comparison with women who had used ADHD medications before pregnancy, suggesting some confounding effect of the underlying ADHD and other associated factors. Our finding of a potential relationship between ADHD medication use during pregnancy and gestational diabetes warrants further investigation, perhaps with greater numbers and capture of additional metabolic risk factors, such as obesity. Overall, it is not possible to determine whether, or to what extent, these findings are an effect of ADHD medication use as opposed to the underlying ADHD.

## Supplementary information

Below is the link to the electronic supplementary material.

Supplementary Material 1

## Data Availability

Direct access to the data and analytical files to other individuals or authorities is not permitted without the express permission of the approving human research ethics committees and data custodians.
