# Supplementary material for: Attention-deficit/hyperactivity disorder medication use and cardiometabolic conditions in pregnancy: a population-based cohort study
Source: Arch Womens Ment Health. 2026 Mar 3;29(2):43. doi: 10.1007/s00737-025-01664-0 (PMC12953407; doi:10.1007/s00737-025-01664-0)
Supplement: Supplementary file 1 — Supplementary Material 1 [file 737_2025_1664_MOESM1_ESM.pdf]

Online resource 1. ICD-10-AM and ACHI codes used to identify childbirth from APDC hospital admission records.

	**Codes to identify childbirth**
**ICD-10-AM code**	O80.0, O80.1, O80.8, O80.9, O81.0, O81.1, O81.2, O81.3, O81.4, O81.5, O82.0, O82.2, O82.8, O82.9, O83.0, O83.1, O83.2, O83.3, O83.4, O83.8, O83.9, O84.0, O84.1, O84.2, O84.8, O84.9, Z37.0, Z37.1, Z37.2, Z37.3, Z37.4, Z37.5, Z37.6, Z37.7, Z37.9, Z38.0, Z38.1, Z38.2, Z38.2 Z38.3, Z38.4, Z38.5, Z38.6, Z38.7, Z38.8
**ACHI code**	90467-00, 90468-00, 90468-01, 90468-02, 90468-04, 90468-06, 90469-00, 90470-00, 90470-01, 90470-02, 90470-03, 90470-04, 90470-05, 16520,00, 16520-01, 16520-02, 16520-03, 16520-04, 16520-05

ICD-10-AM, International Classification of Diseases, Version 10, Australian Modification; ACHI, Australian Classification of Health Interventions; APDC, Admitted Patient Data Collection

Online Resource 2. Graphical depiction of study design.

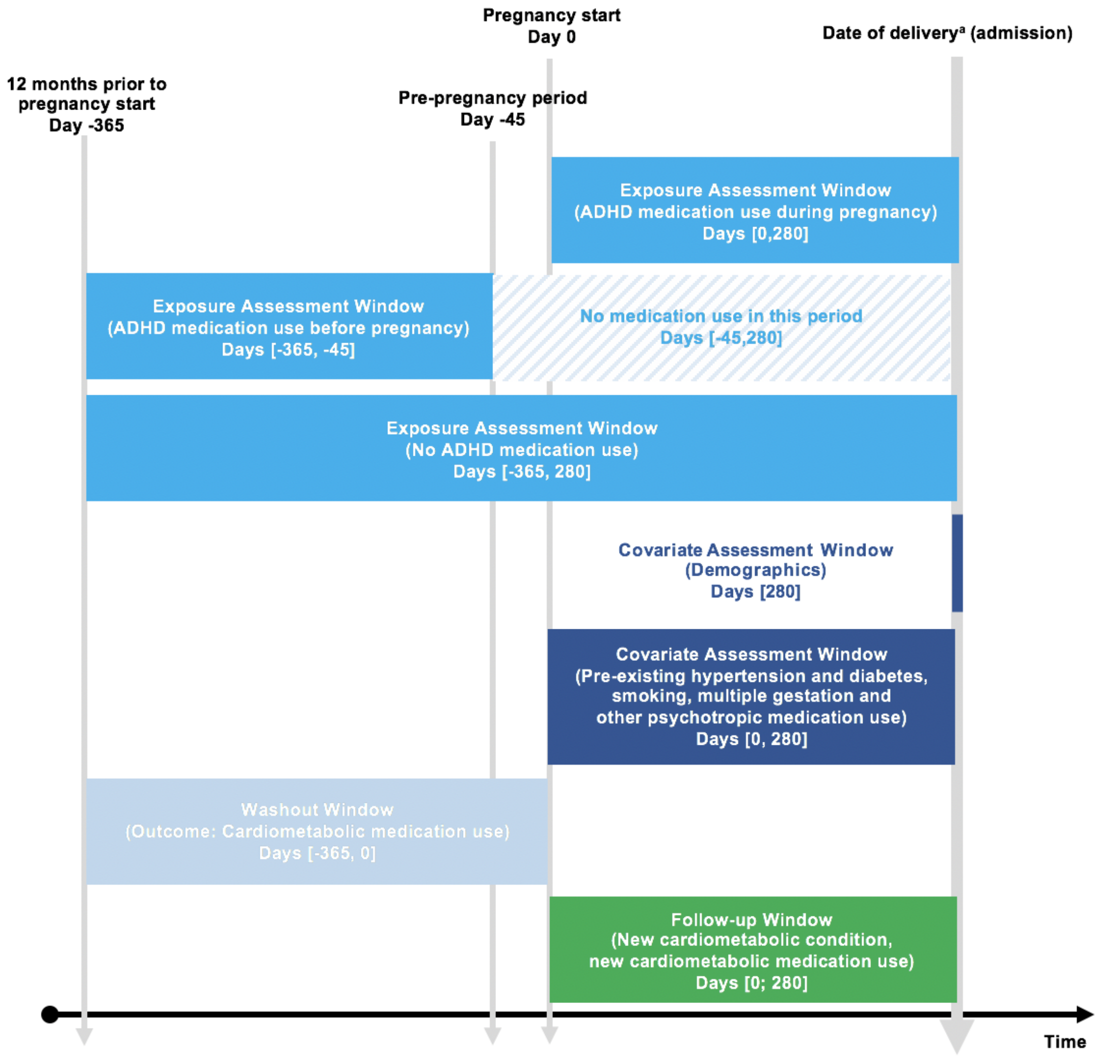

[a] The date of delivery (admission) for a full-term birth of 40 weeks was Day 280. For preterm births, the length of the pregnancy was estimated using diagnosis codes.

Online Resource 3. ICD-10-AM codes used to estimate pregnancy start.

ICD-10-AM code	Estimated pregnancy start
O09.0	Date of delivery minus 5 weeks
O09.1	Date of delivery minus 13 weeks
O09.2	Date of delivery minus 19 weeks
O09.3	Date of delivery minus 25 weeks
O09.4	Date of delivery minus 33 weeks
O09.5	Date of delivery minus 36 weeks
O09.9 or missing	Date of delivery minus 40 weeks

ICD-10-AM, International Classification of Diseases, Version 10, Australian Modification

Online Resource 4. ICD-10-AM codes used to identify cardiometabolic conditions and pregnancy-related characteristics from APDC hospital admission records.

**Condition**	**ICD-10-AM code**
**Gestational hypertension**	O13
**Preeclampsia**	O11, O14.0, O14.1, O14.2, O14.9
**Eclampsia**	O15.0, O15.2, O15.9
**Diabetes mellitus arising in pregnancy**	O24.4, O24.9
**Pre-existing hypertension**	O10.0, O10.1, O10.2, O10.3, O10.4, O10.9
**Pre-existing diabetes mellitus**	O24.0, O24.1, O24.2, O24.3
**Multiple gestation**	O30.0, O30.1, O30.2, O30.8, O30.9, O31.0, O31.1, O31.2, O31.8, O84.0, O84.1, O84.2, O84.8, O84.9, Z37.2, Z37.3, Z37.4, Z37.5, Z37.6, Z37.7, Z37.9, Z38.3, Z38.4, Z38.5, Z38.6, Z38.7, Z38.8
**Smoking**	Z72.0

ICD-10-AM, International Classification of Diseases, Version 10, Australian Modification; APDC, Admitted Patient Data Collection

Online Resource 5. ATC codes for cardiometabolic medications.

Medication	ATC code
**Antihypertensive medications**	C03AA01-C03BA11, C03DB01, C03DB99, C03EA01, C09BA02-C09BA09, C09DA02-C09DA08, C02AB01-C02AC05, C02DB02-C02DB99 (C03CA01-C03CC01, C09CA01-C09CX99), C07AA01-C07AA06, C07AA08-C07AB01, C07AB02, C07AG01, C08CA01-C08DB01, C09DB01-C09DB04, C09DX01, C09BB02-C09BB10, C07AB03, C09DX03, C10BX03
**Antihyperglycaemic medications**	A10

ATC, Anatomical Therapeutic Chemical

Online Resource 6. Study flow chart.

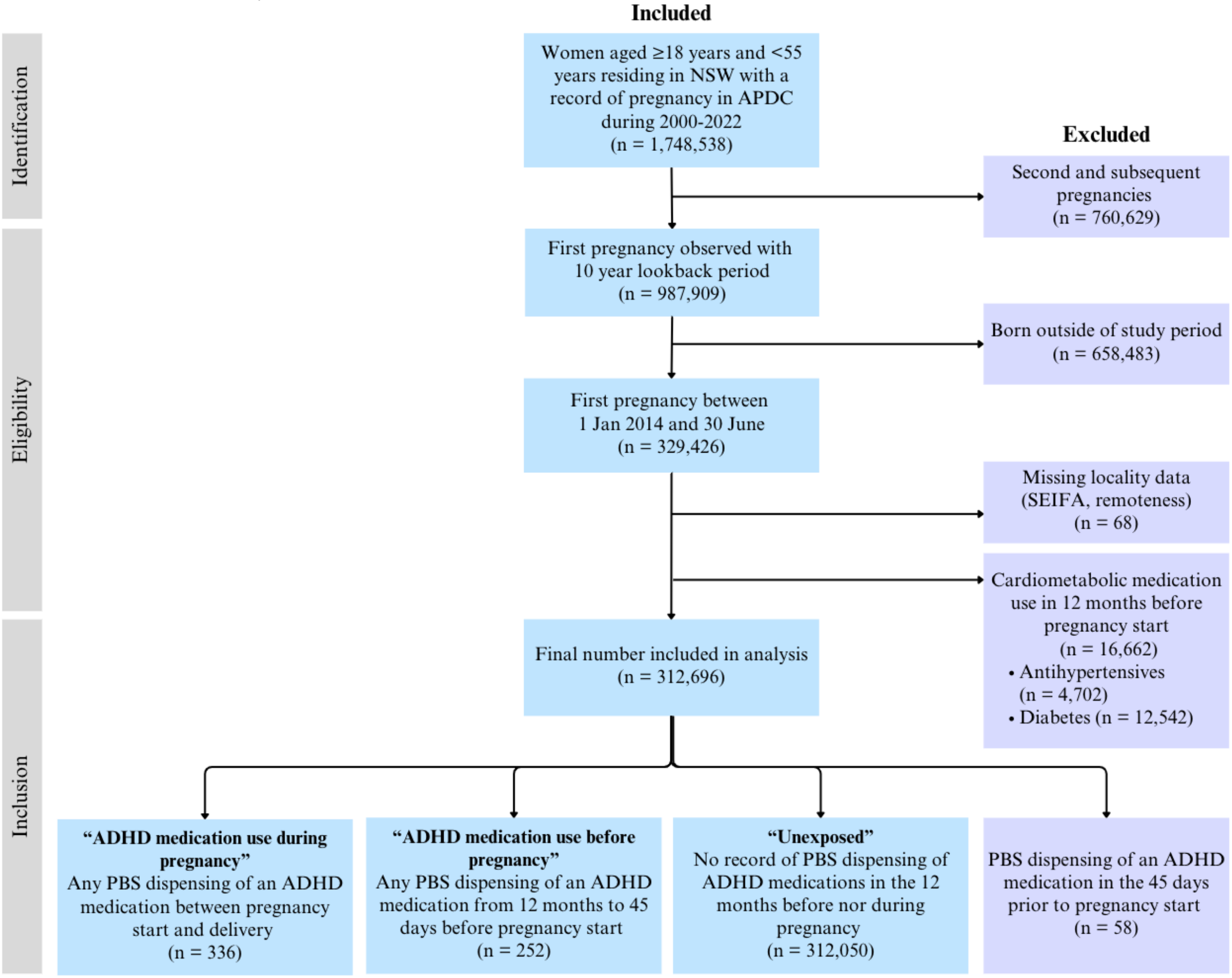

ADHD, attention-deficit hyperactivity disorder; NSW, New South Wales; APDC, Admitted Patient Data Collection; SEIFA, Socio-Economic Indexes for Areas; PBS, Pharmaceutical Benefits Scheme

Online Resource 7. Strengthening the Reporting of Observational Studies in Epidemiology (STROBE) Statement checklist

	Item No	Recommendation	Page No
**Title and abstract**	1	(*a*) Indicate the study's design with a commonly used term in the title or the abstract	1,2
		(*b*) Provide in the abstract an informative and balanced summary of what was done and what was found	
**Introduction**			
Background/rationale	2	Explain the scientific background and rationale for the investigation being reported	3
Objectives	3	State specific objectives, including any prespecified hypotheses	3
**Methods**			
Study design	4	Present key elements of study design early in the paper	4
Setting	5	Describe the setting, locations, and relevant dates, including periods of recruitment, exposure, follow-up, and data collection	4
Participants	6	(*a*) Give the eligibility criteria, and the sources and methods of selection of participants. Describe methods of follow-up	4
		(*b*) For matched studies, give matching criteria and number of exposed and unexposed	5
Variables	7	Clearly define all outcomes, exposures, predictors, potential confounders, and effect modifiers. Give diagnostic criteria, if applicable	4,5
Data sources/ measurement	8*	For each variable of interest, give sources of data and details of methods of assessment (measurement). Describe comparability of assessment methods if there is more than one group	4,5
Bias	9	Describe any efforts to address potential sources of bias	4,5
Study size	10	Explain how the study size was arrived at	4
Quantitative variables	11	Explain how quantitative variables were handled in the analyses. If applicable, describe which groupings were chosen and why	4,5
Statistical methods	12	(*a*) Describe all statistical methods, including those used to control for confounding	5,6
		(*b*) Describe any methods used to examine subgroups and interactions	
		(*c*) Explain how missing data were addressed	
		(*d*) If applicable, explain how loss to follow-up was addressed	
		(*e*) Describe any sensitivity analyses	
**Results**			
Participants	13*	(a) Report numbers of individuals at each stage of study—eg numbers potentially eligible, examined for eligibility, confirmed eligible, included in the study, completing follow-up, and analysed	7
		(b) Give reasons for non-participation at each stage	
		(c) Consider use of a flow diagram	
Descriptive data	14*	(a) Give characteristics of study participants (eg demographic, clinical, social) and information on exposures and potential confounders	7
		(b) Indicate number of participants with missing data for each variable of interest	
		(c) Summarise follow-up time (eg, average and total amount)	
Outcome data	15*	Report numbers of outcome events or summary measures over time	7

Main results	16	(*a*) Give unadjusted estimates and, if applicable, confounder-adjusted estimates and their precision (eg, 95% confidence interval). Make clear which confounders were adjusted for and why they were included	7
		(*b*) Report category boundaries when continuous variables were categorized	
		(*c*) If relevant, consider translating estimates of relative risk into absolute risk for a meaningful time period	
Other analyses	17	Report other analyses done—eg analyses of subgroups and interactions, and sensitivity analyses	7,8
**Discussion**			
Key results	18	Summarise key results with reference to study objectives	9
Limitations	19	Discuss limitations of the study, taking into account sources of potential bias or imprecision. Discuss both direction and magnitude of any potential bias	10,11
Interpretation	20	Give a cautious overall interpretation of results considering objectives, limitations, multiplicity of analyses, results from similar studies, and other relevant evidence	9,11
Generalisability	21	Discuss the generalisability (external validity) of the study results	9,10
**Other information**			
Funding	22	Give the source of funding and the role of the funders for the present study and, if applicable, for the original study on which the present article is based	1

Online Resource 8. Sociodemographic and pregnancy related characteristics of the 1:10 matched unexposed group.

	ADHD medication use during pregnancy N	(%)	Unexposed (matched) N	(%)
**Total, N**	336		3,360	
**Year of childbirth, n (%)**				
2014-15	73	(21.7%)	710	(21.7%)
2016-17	80	(23.8%)	800	(23.8%)
2018-19	83	(24.7%)	830	(24.7%)
2020-21	100	(29.8%)	1,000	(29.8%)
**Maternal age at childbirth (years), mean (SD)**	29.1 (6.2)		29.1 (6.1)	
**Maternal age, years**				
18-24	91	(27.1%)	910	(27.1%)
25-29	95	(28.3%)	950	(28.3%)
30-34	87	(25.9%)	870	(25.9%)
≥ 35	63	(18.8%)	630	(18.8%)
**Maternal country of birth**				
Australia	295	(87.8%)	2,131	(63.4%)
Overseas	41	(12.2%)	1,229	(36.6%)
**Remoteness**				
Major cities	261	(77.7%)	2,607	(77.6%)
Inner regional	58	(17.3%)	538	(16.0%)
Outer regional/Remote	17	(5.1%)	215	(6.4%)
**SEIFA**				
1 – most disadvantaged	65	(19.3%)	797	(23.7%)
2	62	(18.5%)	690	(20.5%)
3	52	(15.5%)	646	(19.2%)
4	77	(22.9%)	630	(18.8%)
5 – least disadvantaged	80	(23.8%)	597	(17.8%)
**Hospital type**				
Public	277	(82.4%)	2,681	(79.8%)
Private	59	(17.6%)	679	(20.2%)
**Marital status**				
Married	219	(65.2%)	2,645	(78.7%)
Unmarried	113	(33.6%)	686	(20.4%)
Missing	4	(1.2%)	29	(0.9%)
**Multiple gestation**	< 6	(< 1.8%)	53	(1.6%)
**Smoking during pregnancy**	35	(10.4%)	6,220	(6.5%)
**Pre-existing hypertension**	< 6	(< 1.8%)	6	(0.2%)
**Pre-existing diabetes**	< 6	(< 1.8%)	< 6	(<0.2%)
**Use of any other psychotropic medication during pregnancy**[a]	148	(44.0%)	386	(11.5%)
**Antidepressants**	114	(33.9%)	180	(5.4%)
**Antipsychotics**	24	(7.1%)	20	(0.6%)
**Antiepileptics**	10	(3.0%)	13	(0.4%)
**Opioids**	41	(12.2%)	205	(6.1%)
**Use of any other psychotropic medication within 12 months prior to pregnancy**	205	(61.0%)	656	(19.5%)
**Antidepressants**	150	(44.6%)	292	(8.7%)
**Antipsychotics**	34	(10.1%)	40	(1.2%)
**Antiepileptics**	16	(4.8%)	21	(0.6%)
**Opioids**	87	(25.9%)	371	(11.0%)

[a] Psychotropic medication categories not mutually exclusive, hence do not add to the total number in each column.
ADHD, attention-deficit/hyperactivity disorder.; SD, standard deviation; SEIFA, Socio-Economic Indexes for Areas
